# Endothelium-Derived Relaxing Factors and Endothelial Function: A Systematic Review

**DOI:** 10.3390/biomedicines10112884

**Published:** 2022-11-10

**Authors:** Francesco Nappi, Antonio Fiore, Joyce Masiglat, Teresa Cavuoti, Michela Romandini, Pierluigi Nappi, Sanjeet Singh Avtaar Singh, Jean-Paul Couetil

**Affiliations:** 1 Department of Cardiac Surgery, Centre Cardiologique du Nord, 93200 Saint-Denis, France; 2 Department of Cardiac Surgery, Hôpitaux Universitaires Henri Mondor, Assistance Publique-Hôpitaux de Paris, 94000 Creteil, France; 3 Department of Clinical and Experimental Medicine, University of Messina, 98122 Messina, Italy; 4 Department of Cardiothoracic Surgery, Royal Infirmary of Edinburgh, Edinburgh EH16 4SA, UK

**Keywords:** endothelial functions, endothelium-derived relaxing factor, nitric oxide, reactive oxygen species, shear stress, perivascular adipose tissue, AMP-activated protein kinase

## Abstract

Background: The endothelium plays a pivotal role in homeostatic mechanisms. It specifically modulates vascular tone by releasing vasodilatory mediators, which act on the vascular smooth muscle. Large amounts of work have been dedicated towards identifying mediators of vasodilation and vasoconstriction alongside the deleterious effects of reactive oxygen species on the endothelium. We conducted a systematic review to study the role of the factors released by the endothelium and the effects on the vessels alongside its role in atherosclerosis. Methods: A search was conducted with appropriate search terms. Specific attention was offered to the effects of emerging modulators of endothelial functions focusing the analysis on studies that investigated the role of reactive oxygen species (ROS), perivascular adipose tissue, shear stress, AMP-activated protein kinase, potassium channels, bone morphogenic protein 4, and P2Y2 receptor. Results: 530 citations were reviewed, with 35 studies included in the final systematic review. The endpoints were evaluated in these studies which offered an extensive discussion on emerging modulators of endothelial functions. Specific factors such as reactive oxygen species had deleterious effects, especially in the obese and elderly. Another important finding included the shear stress-induced endothelial nitric oxide (NO), which may delay development of atherosclerosis. Perivascular Adipose Tissue (PVAT) also contributes to reparative measures against atherosclerosis, although this may turn pathological in obese subjects. Some of these factors may be targets for pharmaceutical agents in the near future. Conclusion: The complex role and function of the endothelium is vital for regular homeostasis. Dysregulation may drive atherogenesis; thus, efforts should be placed at considering therapeutic options by targeting some of the factors noted.

## 1. Introduction

The role of the endothelium in the modulation of vascular tone is crucial because it favours the relaxation of the underlying vascular smooth muscle by releasing mediators of vasodilation. This specific function involves the synthesis and release of endothelium-derived relaxing factor (EDRF), of which the best characterized as nitric oxide (NO). The function of NO is substantially coupled to a synergistic role mediated by vasodilatory prostaglandins and the endothelium-dependent hyperpolarization (EDH). The hyperpolarization of the cell membrane of vascular smooth muscle is primarily orchestrated by EDH. The work of the endothelium in regulating the vasodilation process is completed by endothelial-derived contracting factors [1,2,3,4,5,6,7].

The part of NO is-well defined leading to the activation of soluble guanylyl cyclase in the vascular smooth muscle cells that ultimately determine the production of cyclic guanosine monophosphate (cGMP) initiating relaxation. Recently, in a clearer dynamic, the role of hydrogen peroxide (H_2_O_2_) has become dominant in the vasodilatory function of the endothelium. Other molecules with this function may also be implicated as endothelium-dependent relaxation factors. One of these is pertussis toxin-sensitive Gi “coupling protein” which promotes responses mediated by α2-adrenergic agonists, serotonin, and thrombin. Likewise, a substantial role in endothelium-dependent relaxations is covered by pertussis toxin-insensitive Gq coupling proteins and bradykinin. The role played by adenosine diphosphate is just as crucial [5,6,7]. 

New factors with a pivotal role in EDRF release have emerged, such as insulin and active adiponectin, that has raised a significant biological interest [8,9,10,11,12,13]. Furthermore, in recent years, substantial evidence has provided a better understanding of endothelial cell mechanisms of NO release and how it can be significantly upregulated or downregulated [8,9,10,11,12,13]. The upregulation is induced by oestrogen production, exercise, and dietary factors while downregulation has been observed with oxidative stress, smoking, pollution, and related to the production of oxidized low-density lipoproteins. 

It should also be emphasized that the level of NO decreases with aging and in vascular diseases, such as diabetes and hypertension. Another important requirement concerns the arteries covered by regenerated endothelium, such as coronaries after angioplasty, which selectively leak the pertussis toxin-sensitive pathway for NO release. This state led to a series of events including vasospasm, thrombosis, seepage of macrophages, cell growth, and an inflammatory reaction leading to atherosclerosis. The critical role offered by the release of NO, EDH, and, in particular, H_2_O_2_ in causing vasodilation opposes the contraction process in which endothelial cells work with a significant role. Endothelial cells (ECs) may also favour the contraction of the underlying vascular smooth muscle cells by releasing contraction factors [2,4,5,6,7]. 

Large amounts of contemporaneous evidence are available supporting the contention that most endothelium-dependent sharp rise in contractile strength is caused by the array of vasoconstrictor prostanoids [2,5]. In support of these results, irrefutable evidence has suggested that the action of endoperoxides and prostacyclins is essential for the activation of TP receptors of vascular smooth muscle cells with prostacyclin playing a major role [2,5]. The degree of endothelium-dependent contractions is aggravated in individuals who manifest oxidative stress, aging, spontaneous hypertension, and diabetes where nitric oxide production can be variably impaired. Therefore, endothelium-dependent contractions contribute substantially to reducing endothelium-dependent vasodilatations in the elderly, hypertensive, and diabetics [1,2,3,4,5,6,7,8,9,10,11,12,13].

There are two points that deserve further pondering. In the first issue, findings supported in recent studies have suggested that endothelin-1 release may contribute to endothelial dysfunction. In fact, the peptide appears to be significantly involved in causing vascular dysfunction. The second issue points out the role of nitric oxide itself, which, under certain conditions such as the occurrence of a hypoxic state caused by a reduction in physiological blood flow, can lead to a distorted activation of soluble guanylyl cyclase. This phenomenon evokes, in the underlying vascular smooth muscle, the production of cyclic inosine monophosphate (cIMP) instead of cGMP with a consequential triggered contractile response rather than that of relaxation [2,14].

In the presence of pathological conditions, the functional responses elicited by the maintenance of correct homeostasis of NO, EDH, and in particular H_2_O_2_, in addition to endogenous hyperglycemic hormones, are advantageous in order to ensure the correct maintenance of vascular tone [5,8,9,10,11,14]. Likewise, the development of a state of vasoconstriction, prothrombotic and proinflammatory may occur in cases where one of the responses mediated by a vasoactive factor is compromised to varying degrees [5].

The concept that endothelial dysfunction is primarily determined by a reduction in the production or action of relaxing factors derived from the endothelium is well-established. Likewise, the persistence of this state could be configured as the primary driver for the evolution of cardiovascular disease.

Concerns about changing endothelial functions in humans have prompted physicians to consider some surrogates as crucial markers of cardiovascular events. In this way, potential markers of endothelial dysfunction have been highlighted in clinical practice and represent an open window toward the onset of future cardiovascular disorders and events related to coronary heart disease. An example of endothelial dysfunction is revealed by the reduced flow-mediated dilatation of the brachial artery or the digital reactive hyperaemia index in peripheral arterial tonometry [15,16,17]. Individuals who disclose a 1-SD (source determination) decrease in flow-mediated dilation or reactive hyperaemia index are associated with a doubled risk of cardiovascular events [18]. Therefore, a correct clinical observation of these patients is of fundamental importance, suggesting that the endothelial function in the peripheral vascular beds could be a predictor of potential cardiovascular events.

Although arteriosclerosis, thrombosis, and vascular biology have been studied for more than 40 years, detailed current advances and trends in the research on endothelial functions deserve a thorough analysis, because knowledge is constantly evolving and new evidence has recently emerged [14]. Moreover, the current biological and biochemical research demonstrates a clear contradiction between the proven benefits of statins administration as compared to very questionable improvement in the evolution of atherosclerotic lesions for patients who were managed with percutaneous coronary intervention in everyday clinical practice [19,20,21].

To encourage a better understanding of the residual risk of progression of primary atherosclerotic lesions for patients and to provide guidance for clinicians, here we discuss the current evidence base regarding endothelial function and the mechanisms involved in vasodilation. 

## 2. Methods

### 2.1. Search Strategy

In March 2022, PubMed, Ovid’s version of MEDLINE, and EMBASE database were searched using the terms “Endothelial vasodilation (5202 to the present)” “Endothelial vasoconstriction (1102 to the present)” “nitric oxide (18,810 to the present)”, “endothelium-derived relaxing factor (18,908 to the present)”, “hydrogen peroxide (9770 to the present)” coupled with “atherosclerosis”, and “endothelial function,” (more than 54,799 to the present). This comprehensive search identified data from basic research articles and randomized controlled trials (RCT). The review was registered with the OSF register of systematic reviews, and followed the Preferred Reporting Items for Systematic Reviews and Meta-analyses (PRISMA) reporting guidelines. 

### 2.2. Study Selection and Data Extraction

The searches retrieved 6520 relevant abstracts, and, after deduplication, 5842 relevant citations were screened by 3 reviewers on their own (A.F. J.M, PL. N). Inconsistency was resolved by a fourth author (F.N). The predefined inclusion criteria settled the stage for reviewing the titles and abstracts. The articles included were in English and were very impressive research articles based on the function of NO, EDRF, and H_2_O_2_; additionally of great relevance were the RCTs focused on polymorphism of endothelial nitric oxide synthase and observational study on the activity of nicotinamide adenine dinucleotide phosphate (NADPH) oxidase, ROS production, and microvascular endothelial dysfunction in obesity. Relevant animal studies were included because they were of higher impact on the emerged role of endothelial dysfunction in supporting cardiovascular disease. The most relevant articles performed on the animal models were published in Arteriosclerosis, Thrombosis, and Vascular Biology journal. Any case reports, conference presentations, editorials, and expert opinions were excluded.

### 2.3. Endpoints and Effect Summary

The endpoints evaluated the effects of emerging modulators of endothelial functions, focusing the analysis on studies that investigated the role of ROS, perivascular adipose tissue, shear stress, AMP-activated protein kinase, potassium channels, bone morphogenic protein 4, and purinoceptor 2 (P2Y2) receptor. 

## 3. Results

**Figure 1:**
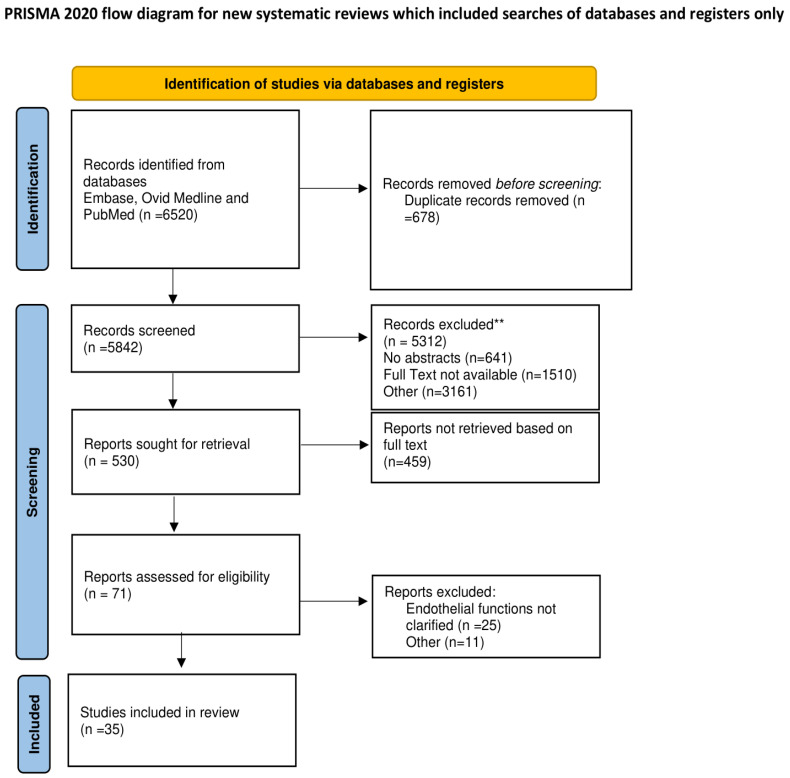
PRISMA 2020 flow diagram for new systematic reviews which included searchers of databases and registers only. From [MJ, McKenzie JE, Bossuyt PM, Boutron I, Hoffmann TC, Mulrow CD, et al. The PRISMA 2020 statement: an updated guideline for reporting systematic reviews. BMJ 2021; 372: n71. doi: 10.1136/bmj. n71].

A total of 530 citations were reviewed, of which 35 studies were included in the final systematic review reaching the eligibility criteria. The endpoints were evaluated in these studies, which offered an extensive discussion on emerging modulators of endothelial functions. The full PRISMA flow diagram and checklist outlining the study selection process are reported both in Figure 1 and in Supplementary Table S1. The details of the studies are included in Table 1, Table 2 and Table 3 of the related chapters described along with the full illustration of the results with related references. 

### 3.1. Reactive Oxygen Species

Here, we describe the role of Reactive Oxygen Species (ROS) which have been considered primarily harmful due to their highly noxius structure to cells and tissues. Furthermore, they can have pathological implications in a wide range of cardiovascular diseases and endothelial dysfunctions. Many studies consider the physiological roles of ROS both in animal models and for patients with coronary artery disease (CAD); however, this reveals a striking contrast between the favourable regulation that emerged in some studies on vascular homeostasis versus the development of severe tissue damage found in other studies (Table 1).

**Table 1:** Studies reporting the effect of ROS species. The references represent the progression in the text according to those reported in the PRISMA flowchart.

First Author/Year Ref	Type of Study	Cohort	Aims	Findings
Wang et al. 2017 JAHA [22]	Rat Model	SHR WKY Control	TRPC3 induced ROS production induced.	Improved TRPC3 activity at the cytoplasmic and mitochondrial levels. Increased redox signaling and calcium dysregulation.
Montezano et al. 2016 JAHA [23]	Mice model	SM22^+^, Nox5^+^, Nox5^+^/SM22^+ †^ WT	Nox5 in pro-contractile signaling and vascular function.	Nox5 lead to join calcium and reactive oxygen species to the pro-contractile molecular apparatus in vascular smooth muscle cells.
Ikumi et al. 2020 J Cardiovasc Pharmacol [24]	Mice model	eNOS-KO nNOS/eNOS-double-KO WT control	EDH in coronary microcirculation and cardiac diastolic function.	Substantial effect of EDH/H_2_O_2_ in maintaining coronary microcirculation and cardiac diastolic function through oxidative PKGIα activation.
Denhartig et al. 2017 Arterioscler Thromb Vasc Biol [25]	Mice model	C57BL/6 NOX4 deletion C57BL/6 control	NOX4-derived ROS in the development of obesity and insulin resistance.	Substantial role of NOX4-derived ROS in the onset of insulin resistance and adipose tissue inflammation.
Sano et al. 2017 Nutr Metab Cardiovasc Dis [26]	Mice model	C57BL/6 HFD C57BL/6 HFD plus EC	In vivo effects of EC on adipose tissue inflammation and obesity.	Noticable beneficial effects of EC for the prevention of adipose tissue inflammation and insulin resistance by marked suppression of CCL19.
Li et al. 2022 [27] PLoS One	Rat Model	AT1aR−/− gene KO WT control	The role of Ang II in diet-induced obesity.	AT1aR deficiency alleviated adipocyte hypertrophy in high-fat diet rats by promoting adipose lipolysis probably via cAMP/PKA pathway.
La Favor et al. 2016 Arterioscler Thromb Vasc Biol [28]	Human model	Men 15 Women 27 BMI 21.6 ± 0.6 30.1 ± 0.4 36.6 ± 0.7 ℷ	Determination the impact of in vivo ROS on microvascular endothelial function in obese human individuals.	Substantial increase activity of NADPH oxidase. Excessive ROS production in skeletal muscle of obese individuals. Association between excessive NADPH oxidase-derived ROS to microvascular endothelial dysfunction in obesity.
Masi et al. 2018 Arterioscler Thromb Vasc Biol [29]	Human model	36 obeses 31 controls	Impact of arginase as a determinant of endothelial dysfunction in small arteries in obese individuals.	Arginase lead to microvascular endothelial dysfunction in obesity. Decreased effect of arginase in aged obese for higher levels of vascular oxidative stress. Accelerated microvascular remodeling in obese individuals.
Godo et al. 2016 Arterioscler Thromb Vasc Biol [30]	Mice model	Cav-1-KO eNOS-Tg WT	Study the probable relevance of the physiological balance between NO and EDH in cardiovascular homeostasis.	Genotypes showed altered cardiovascular phenotypes, including cardiac hypertrophy in Cav-1-KO mice and hypotension in eNOS-Tg mice. Evidence suggested that excessive endothelium-derived NO with reduced EDH impairs cardiovascular homeostasis in mice in vivo.
Gray et al. 2016 Arterioscler Thromb Vasc Biol [31]	Human model Mouse model	HAECs_ diabetic HAECs_ non diabetic	Investigate the role of type 4 isoform (NOX4) in human and mouse atherosclerosis.	Both in humans and in mouse, the H_2_O_2_ -forming NOX4, in contrast with the superoxide-forming NOX1, can act as a negative modulator of inflammation and remodeling and convey atheroprotection.
Otsuka et al. 2013 Ann Cardiothorac Surg [32]	Human model	Comparative study IMA vs. SVG	IMA reveals fewer fenestrations, lower intercellular junction permeability. Higher antithrombotic production of heparin sulfate and tissue plasminogen activator, and NO.	IMA is the first line of defense for the treatment of coronary artery disease.
Freed et al. 2014 Circ Res [33]	Human model	Human healthy adipose arterioles pretreated with ceramide	To evaluate the induction of ceramide to favor a switch from nitric oxide to H_2_O_2_.	Ceramide has an crucial role in the transition from nitric oxide to mitochondrial-derived H_2_O_2._
Durand et at 2016 Arterioscler Thromb Vasc Biol [34]	Human model	Patients with CAD	To evaluate vascular actions of angiotensin 1-7 (ANG 1-7) in human atrial and adipose arterioles.	ANG 1-7 treatment is sufficient to restore the NO component of FMD in arterioles from patients with CAD.
Kirsch et al. 2016 Arterioscler Thromb Vasc Biol [35]	Mouse model	Mouse Cremaster vs. Txnrd2-deficient mice	To examine dysregulated redox homeostasis and inadequate nitric oxide signaling.	Txnrd2 plays a crucial important role in balancing mitochondrial ROS production in the endothelium.
Erb et al. 2016 Arterioscler Thromb Vasc Biol [36]	Human model	Patients (n = 51) undergoing CABG with IMA	To evaluate both polymorphisms of nitric eNOS gene in the promoter (T-786C) and exon 7 (G894T).	Observed eNOS polymorphisms in larges conduits. Deteriorated endothelium-dependent vasodilatory capacity in patients with CAD.

Abbreviations: ANG 1-7, angiotensin 1-7; Angiotensin II receptor type 1, AT1aR−/−, AT1aR gene knockout; CABG, coronary artery bypass grafting; Cav-1, caveolin-1; EC, Epicatechin; EDH, endothelium-dependent hyperpolarization; eNOS, endothelial NOS; Endothelium-derived hydrogen peroxide; H_2_O_2_; FID, flow-induced dilation; HAEC, human arterial endothelial cell; HDF, high-fat diet; IMA, internal mammary artery; KO, knockout; NADPH, nicotinamide adenine dinucleotide phosphate; NOX, NADPH oxidase; nNOS, neuronal NOS; NOS, nitric oxide synthases; PKGIα, protein kinase G I-α; ROS, reactive oxygen species; SHR, spontaneously hypertensive rat; SM, smooth muscle; Tg, transgenic; TRPC3; transient receptor potential channel, canonical type 3; WKY, wistar kyoto rat; WT, wild type; †22, protein type; ℷ obese.

**Figure 2:**
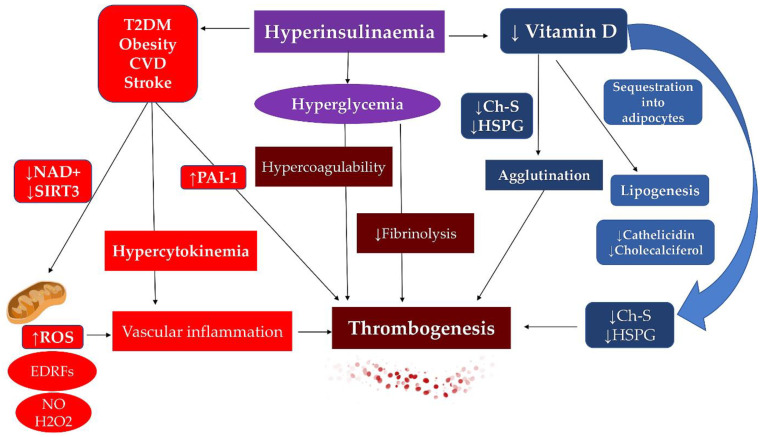
Endothelial dysfunction is orchestrated by various mechanisms: metabolites, molecular and inflammatory. Hyperinsulinemia, CVD and vitamin D have a strong impact on homeostatic equilibrium. Both hyperinsulinemia and hyperglycaemia generate states of increased coagulation and decreased fibrinolysis. By driving the development of CVD, diabetes mellitus and obesity, they contribute to the inflammatory substrate of cytokines. They increase the ROS production due to the damage in decreasing both NAD+ and reduced glutathione (GSH). A reduction in vitamin D due to sequestration into the adipocytes induces lipogenesis with de-creased level of cathelicidin and cholecalciferol, leading to decreased levels of ChS and HSPG, regulators of RBCs deformation and increased cells agglutination. These mechanisms are all responsible for thrombosis initiation. The vascular inflammation and decreasing levels of Ch-S and HSPG are represented with coloured backgrounds being the main actors of thrombogenesis trigger. In addition, thrombogenesis, the main effect, is outlined with a different colour too. Abbreviations: Ch-S: cholesterol sulfate, CVD, cardiovascular disease; EDRFs, endothelium-derived relaxing factors, HSPG: heparan sulfate proteoglycans, NAD+: nicotinamide adenine dinucleotide, PAI-1 plasminogen activator inhibitor type 1, RBC, red blood cell; ROS: reactive oxygen species, T2DM: type 2 diabetes mellitus, SIRT3: sirtuin 3, TNF-α: tumour necrosis factor-α, IL-6: interleukin 6.

Several independent studies demonstrated significant harm by the production of ROS. These molecules play a crucial role in tissue injury due to their substantially detrimental effect on cells and tissues leading to pathological implications that reveal themselves in a wide range of cardiovascular diseases, vascular smooth muscle cells, and endothelial dysfunctions [22,23,24,37,38,39] (Figure 2).

We learned that endothelial dysfunction is the stamp of authentication of atherosclerotic cardiovascular diseases. It should be noted that the contribution of EDRFs to endothelium-dependent vasodilatation fluctuates, with a distinct mode that is related to the vessel size. For example, NO mainly lead vasodilatation in relatively large vessels, as was highlighted for epicardial coronary arteries, while EDH factors play a preferential role in small resistance vessels such as coronary microvessels. On the other end, endothelium-derived hydrogen peroxide (H_2_O_2_) serves as one of the crucial EDH factors mostly in microcirculations, representing a pivotal physiological signalling molecule. H_2_O_2_ has gained increasing consideration for its emerging relevance in cardiovascular diseases and cardiac diastolic function through oxidative protein kinase G I-α (PKGIα) activation [24].

The transient receptor potential channel, canonical type 3 (TRPC3), which is expressed in the mitochondria, has the distinctive role to ensure mitochondrial calcium homeostasis [22]. Evidence based on the spontaneously hypertensive rats (SHRs) model revealed that the inhibition of TRPC3 attenuates vascular calcium influx, the latter depending on the ROS production levels. The results by Wang et al. [22] revealed an enhancement of TRPC3 activity at the cytoplasmic and mitochondrial levels which contributes to redox signalling and calcium dysregulation in the vascular system by SHR. Substances such as angiotensin II or drugs such as telmisartan can regulate mitochondrial [Ca^2+^], levels of ROS production, and mitochondrial energy metabolism by selectively acting on TRPC3 [22]. Montezano et al. [23] defined a new function for vascular NADPH oxidase 5 (Nox5) starting from the peculiarity of its distribution. Rodents do not express Nox 5 which is instead present in the lower forms and higher mammals. Nox5 generates calcium-sensitive superoxide with a pro-contractile effect in vascular smooth muscle cells. In particular, Nox5 is a pro-contractile Nox isoform important in redox-sensitive contraction. This action plays a crucial role in the regulatory mechanisms of the endoplasmic reticulum and calcium-calmodulin. Researchers highlighted the specific role of vascular Nox5, which relates reactive calcium and oxygen species to the pro-contractile molecular structure in vascular smooth muscle cells.

The development of obesity is also related to increased ROS levels as disclosed using adipocyte-specific NADPH (nicotinamide adenine dinucleotide phosphate) oxidase 4–deficient mice. The findings from Den Hartigh et al. suggested [25,40] that adipocyte NADPH oxidase 4–derived ROS favoured the development of insulin resistance-related obesity by precipitating inflammation of adipocytes. Likewise, inflammation of the adipose tissue is one of the main causes that leads to an increased risk for coronary heart disease. Epicatechin has been shown to exert beneficial effects for the prevention of inflammation of adipose tissue and insulin resistance. Sano et al. [26,41], based on a previous study that reported that protection from diet-induced obesity and insulin resistance in mice deficient in chemokine (CC motif) ligand 19 (CCL19) expression led to a deficiency of CCL19 receptor, suggested that the beneficial effects of Epicatechin could be induced, at least in part, by the noticeable suppression of CCL19 expression.

These results were recently confirmed in the Angiotensin II receptor type 1 (AT1aR) gene knockout rat model, which was used to study the special role of Ang II on adipose lipid metabolism. Evidence reported that the renin-angiotensin system (RAS) was overactivated. Furthermore, the serum levels of angiotensin II (Ang II) were increased in obese patients, suggesting that an AT1aR deficiency had relieved adipocyte hypertrophy in rats on a high-fat diet by promoting adipose lipolysis probably via cAMP/PKA pathway [27].

ROS levels in obese subjects were measured with a new microdialysis technique which, in addition to enabling simultaneous measurement of ROS levels, also allowed the evaluation of microvascular endothelial functions in vivo. Using this method, the investigators reported increased levels of ROS in obese individuals that were derived from NADPH oxidase. Endothelial dysfunction, with the associated excessive production of oxidizing enzyme, is expressed through an altered response to acetylcholine, which would reduce blood flow. The relevant data demonstrated in this study underline that an 8-week aerobic training normalized both elevated ROS levels and induced normalization of microvascular endothelial dysfunction [28]. Masi et al. [29] studied the effect of arginase as a determinant of endothelial dysfunction in small arteries of obese individuals, focusing on its relationship with aging and microvascular remodelling. In this case, 36 obese patients and 31 controls underwent the dissection of small arteries after subcutaneous fat biopsies that were evaluated on a pressurized micromyograph. The investigators used the media-lumen ratio and amount of vascular wall fibrosis as markers of vascular remodelling. Instead, acetylcholine was used for the assessment of endothelium-dependent vasodilation. This test was repeated under L-NAME (N ^G^-nitro-L-arginine-methyl ester), N(ω)-hydroxy-nor-l-arginine (arginase inhibitor), and gp91ds-tat (NADPH [nicotinamide adenine dinucleotide phosphate oxidase] oxidase inhibitor) in vessels from young (age < 30 years) control, and obese and old (>30 years) control and obese individuals. The evidence revealed lower vascular NO levels for the elderly and obese groups, as well as a higher mean-to-lumen ratio, wall fibrosis, intravascular superoxide, and blunt inhibitory effect of L-NAME on acetylcholine for the elderly and obese groups, compared to controls and younger age groups. Conversely, the effect of N(ω)-hydroxy-nor-l-arginine directly reinstated the acetylcholine-induced vasodilation in young and, to a lower degree, in old obese individuals. In conclusion, although arginase worked to induce microvascular endothelial dysfunction in obesity, its impact was decreased in aged individuals due to higher levels of vascular oxidative stress that occurred. It is important to note that patients with obese conditions revealed an accelerated microvascular remodelling process, the entity of which was substantially related to the levels of arginase present in the vascular wall [29].

The physiological key roles exerted by ROS levels in the regulation of vascular homeostasis were established in a landmark paper from Godo et al. [30,38]. The worsened cardiovascular homeostasis in mice was almost certainly due to its excessive endothelial NO production. The investigators experienced that either a lack of an unfavourable regulator of endothelial NO synthase (eNOS) caveolin-1 or overexpression of eNOS disturbed (deranged) the physiological balance between NO and H_2_O_2_ as an EDH factor in microcirculations. These peculiar functional characteristics of eNOS probably explain the impaired cardiovascular homeostasis in mice. In fact, both genotypes of Cav-1-knockout (Cav-1-KO) and endothelium-specific eNOS transgenic (eNOS-Tg) mice disclosed altered cardiovascular phenotypes, including cardiac hypertrophy in Cav-1-KO mice and hypotension in eNOS-Tg mice [30]. Likewise, the NADPH oxidase 4–derived hydrogen peroxide (H_2_O_2_) has an atheroprotective role in a mouse model that develops diabetic atherosclerosis and in the human internal thoracic artery, disclosing multiple peculiar interventions both on elastic laminae and on specific muscular components, which explains its reduced tendency for spasms and the development of atherosclerosis [31,32]. Otsuka et al. [32] revealed that as compared with all other arterial and venous conduits, the human internal thoracic artery experienced an increased production of anti-inflammatory and vasoactive molecules, particularly nitric oxide.

Gutterman’s group [42] worked on an untested mechanism of microvascularisation in order to explain the phenomena that support dysfunction in human coronary artery disease. Researchers have shown that proper and functional human coronary circulation is regulated by NO and low physiological levels of H_2_O_2_ as an EDH-related factor. However, the incidence of serious atherosclerotic conditions and metabolic disorders can be significantly related to a switch from NO to H_2_O_2_ as the influencer for endothelium-dependent relaxations. Therefore, consideration should be given to the higher pathological levels of H_2_O_2_ as responsible either for microvascular dysfunction or for the development of coronary artery disease [42]. Once more, the same group of investigators revealed crucial evidence on the emerging role of the ceramide-induced reduction of telomerase activity in mitochondria as determinants in causing a switch from NO to H_2_O_2_ [33,34].

From a physiological point of view, findings supporting the mechanisms that regulate the level of ROS have not provided conclusive evidence. However, the evidence available suggests a predominant role of local subcellular ROS concentrations in microdomains rather than a role played by absolute intracellular concentrations. The former can be fundamental in determining whether the effects of ROS cause useful or harmful outcomes for cellular processes. Moreover, the colocalisation of the ROS source and target seems to play a key role in preventing non-specific noxious oxidations [43]. Two independent studies have highlighted the substantial role of two redox proteins in endothelial healthy homeostasis [35,44]. The first is endothelial thioredoxin reductase 2, which plays a substantial role in preserving the healthy functions of the endothelium [35]. The second is the peroxisome proliferator receptor-γ coactivator 1α, which has been suggested as the primary regulator of endothelial functions including protection against oxidative stress, inflammation, and atherosclerosis [44]. In conclusion, the mechanism of action of ROS explains an apparent double physiological role that they play; thus, proving to be significant endogenous signalling molecules. This well-defined role opens a window in the field of the development of the best therapeutic strategies aimed at reducing the level of pathological ROS, such as in the presence of isoform or site-specific inhibitors of NADPH oxidase [14].

The role of NO is pivotal to assure graft permeability in patients who received coronary bypass grafting (CABG) surgery with the use of left internal thoracic mammary (LITA) anastomosed on the left anterior descending artery. The impact of both polymorphisms of the eNOS gene in the promoter (T-786C) and exon 7 (G894T) on the ex-vivo vasomotor function of LITA, used as a conduit for first target vessel revascularization, were investigated in 51 consecutive patients with coronary artery disease (CAD) undergoing CABG operation. Mammary artery rings were achieved, and endothelium-dependent ring relaxation was determined in vitro in an organ chamber using acetylcholine (10^−9^ M and 3 × 10^−4^ M). The LITA is the arterial conduit for which there is the greatest derived evidence of endothelium-dependent relaxation evaluated in vitro, and where an alteration in those positive for the T-786C or the G894T eNOS polymorphism has been significantly reported. This evidence suggests that the presence of either one of the eNOS polymorphisms leads to a compromise of the endothelium-dependent vasodilatory capacity of large conduit vessels; thus, representing an additional factor to the classical risk factors in patients with CAD [36].

### 3.2. Shear Stress

Biologists and fluid dynamics experts have taught us that flow is the marker of living. 

**Figure 3:**
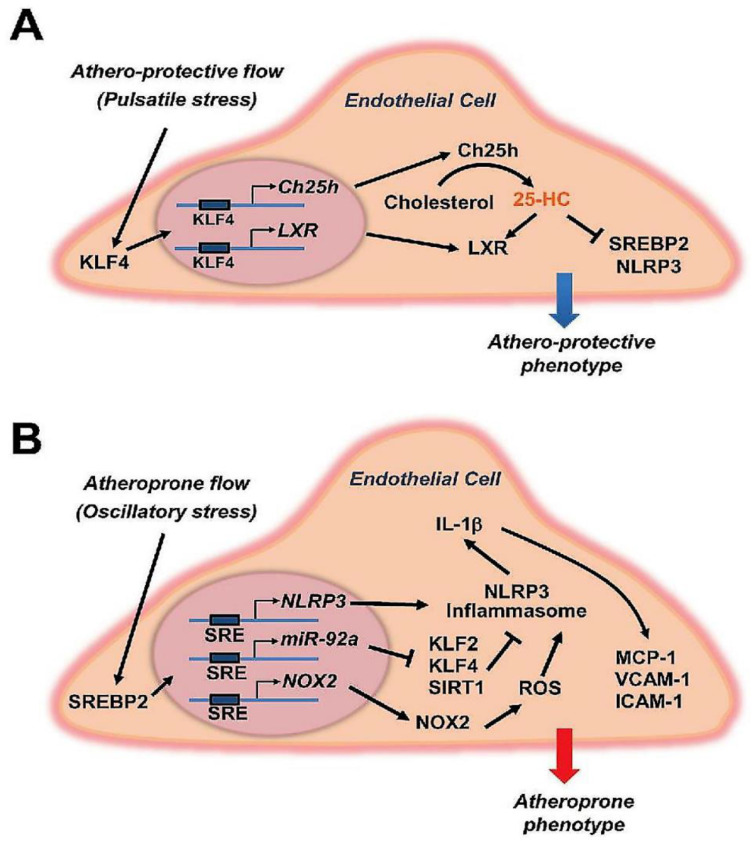
Fluid dynamics regulate the function of endothelial cell through (**A**) an atheroprotective effect favoured by the pulsatile flow inducing a higher expression of Kruppel-like factor 4 (KLF4) or alternatively (**B**) an atheroprone effect related to the oscillatory flow that causes an increased level of sterol regulatory element-binding proteins (SREBP). Different biochemical and molecular mechanisms regulate the two different conditions. Atheroprotective flow is characterised by substantial greater production of KLF4 that leads to the expression of Ch25h and LXR with increased intracellular levels. This mechanism promotes higher production of 25-HC, SREBP2 and NLRP3. Atheroprone phenotype as consequence of an atheroprone flow (oscillatory stress) led to increased level of SREBP2 due to greater expression of SRE and its encoding molecules. This nuclear activation favours a detrimental effect by increased intracellular level of KLF2, KLF4, SIRT1 (gene miR-92a), NOX2 (gene NOX2) and NLRP3 inflammasome (gene NLRP3). The results are higher level of IL-1β leading to a major production of MCP-1, VCAM-1 and ICAM-1. Abbreviations: 25-HC = 25-hydroxycholesterol, CH25H = Cholesterol 25-Hydroxylase, KLF = Kruppel-like factor, ICAM-1 = Inter Cellular Adhesion Molecule-1, IL = interleukine, (MCP-1/CCL2) = Monocyte chemoattractant protein-1, NLRP3, NOD-like receptor family, pyrin domain containing 3, SREBP2 = sterol regulatory element-binding protein-2, SIRT1 = Sirtuin 1, VCAM-1 = vascular cell adhesion molecule 1.

The stress imparted by this physical force to the endothelium is translated by the cells, which line its internal surface, into a multitude of signals that regulate the functions of the entire cardiovascular system. This fine mechanism allows for the responses to be regulated in a very precise way to meet physiological demands, in contrast with the other organs. Therefore, shear stress has the substantial role of delivering important physiological signals to endothelial cells, through endothelial mechanotransduction mechanisms that lead ECs to synthesis and release of relaxing factors derived from the endothelium. Likewise, shear stress-induced endothelial NO release not only provides local blood pressure control, but also effectively works to delay the atherosclerotic process [45,46,47,48,49,50,51]. All these determinants promote relaxation of the underlying vascular smooth muscle and vasodilation. The discoveries that have explained the most recent mechanisms of endothelial mechanotransduction open an important window on the development of cardiovascular diseases and how they can be addressed for the health of individuals [45,46,48,49,50,51] (Figure 3).

The background polymorphisms in the promoter (T-786C) and exon 7 (G894T) of the endothelial nitric oxide synthase (eNOS) gene associated with reduced vascular NO production or increased proteolytic cleavage of eNOS were established in a landmark RCT from Erbs et al. [45]. The authors mainly reported the effects of these polymorphisms on endothelial function and endothelial response to physical exercise in 67 enrolled patients with coronary artery disease (CAD) and randomized to either a training or a control group. The acetylcholine was used to induce changes in the average peak velocity (APV) of a coronary or mammary artery and was invasively assessed by Doppler velocimetry at the beginning and after 4 weeks. In the initial phase, individuals with the wild-type (WT) variant revealed an increase in APV in response to acetylcholine. This response was significantly attenuated in patients who were positive for promoter (44 +/− 7%) or exon 7 (62 +/− 9%) polymorphism. After four weeks of physical training, patients experienced an endothelium-dependent increase in APV reaching +36 +/− 12% in promoter polymorphism-positive patients. Conversely, patients with variant WT—and those positive for exon 7 polymorphism recorded an increase of +81 +/− 18% and +91 +/− 15%, respectively. These results suggested that the occurrence of one of the polymorphisms substantially mitigated endothelium-dependent vasodilation in patients with CAD. It is important to note that it was reported that only promoter polymorphism could have a negative effect on training-induced improvement in endothelial function [45].

As Cattaruzza et al. [46] described, the shear stress-induced endothelial nitric oxide (NO) successfully influences a delay in atherosclerosis development. The authors evaluated functionally relevant polymorphisms in endothelial NO synthase (NOS-3) and by focusing on polymorphisms in NOS-3 they demonstrated how this condition can contribute to the development of CAD. The investigators analysed NOS-3 expression in endothelial cells isolated from umbilical cords genotyped for the −786C/T single nucleotide polymorphism (SNP) of the human nos-3 gene. In addition, NO-dependent relaxation was evaluated in rings of saphenous vein isolated from genotyped patients, who received coronary artery bypass grafting operations. Patients subjected to quantitative coronary angiography were genotyped to verify an association between this SNP and CAD. Although evidence revealed that shear stress-induced NOS-3 mRNA and protein expression was found in TT and CT genotype cells, the discovery of NOS-3 mRNA and protein expression were not recorded in cells with the CC genotype. By pre-treating these cells with a decoy oligonucleotide comprising positions −800 to −779 of the C-type nos-3 promoter, the shear stress-induced expression of NOS-3 was reconstituted. The authors subsequently validated these results by performing an analysis of the reporter gene with the corresponding constructs of the luciferase of the nos-3 promoter. It should be noted that the NO-mediated relaxing response of venous grafts from patients with the CC genotype was significantly mitigated as compared to that of individuals with the CT or TT genotype expression. Furthermore, in patients who experienced CAD, the CC genotype was significantly more recurrent than in CHD-negative patients (19.0% vs. 4.4%, respectively). The relevant discovery provided by Cattaruzza’s study suggests that the SNP −786C/T of the nos-3 gene constitutes a genetic risk factor for CHD, presumably due to the binding of an inhibitory transcription factor to the type C promoter that blocks the maintenance of NOS-3 expression dependent on shear stress [46].

The different actions exerted by shear stress on the vessel wall functionality were also established in two other landmark papers [48,49]. One study suggested that the distinct roles of laminar or pulsatile shear stress may induce an atheroprotective effect. Contrary to the effects of oscillatory shear stress, or disturbed flow, which instead revealed an atheroprone effect supported by crucial molecular mechanisms; the latter can be traced back to the patterns of flow previously mentioned [49]. Instead, the evidence reported by Abe and Berk [48] has further reinforced the current knowledge on the dual roles of shear stress, highlighting the effects of the 2 distinct types of flow. If on the one hand, the constant laminar flow supplies atheroprotective effects on the vascular wall by increasing the endothelial production of prostacyclin and NO, whereas, in disturbed flow, there is stimulation that induces proinflammatory signalling. This mechanism supports endothelial dysfunction with the consequent evolution towards the development of atherosclerotic lesions [48].

Two independent studies provided the same findings, thus highlighting the detrimental action of the disturbed flow [50,51]. The first study reported the effects of flow on EC senescence using mice model LDLR (−/−) (low-density lipoprotein receptor (−/−)) that were exposed to a high-fat diet. By means of en face staining, investigators revealed that both senescence-associated β-galactosidase activity and p53 expression were higher in endothelial cells located at disturbed flow sites in response to a high-fat diet. On the contrary, ECs, which were located in protected sites with undisturbed flow, did not express senescence-associated β-galactosidase or p53. These findings proved that disturbed flow was comparable to that observed in vascular sites prone to atherosclerosis, such as arterial branches, bifurcations, and curves, accelerated endothelial senescence through a p53-p21-dependent pathway. The findings offered in these experimental conditions disclosed that the activation of sirtuin-1 using resveratrol or SRT1720 had a protective role against the flow-induced endothelium senescence [50]. The second recent study suggested that endothelial mechanotransduction mechanisms work noticeable roles in the therapeutic angiogenic effects of pulsed ultrasound (LIPUS) [51]. Since LIPUS therapy has been proven effective in myocardial ischemia in a pig model of chronic myocardial ischemia through enhanced myocardial angiogenesis, its role has been suggested to favour an improvement of LV remodelling after acute myocardial infarction. The evidence reported by Shindo et [51] al proved that LIPUS induced an upregulation of the expression of vascular endothelial growth factor, endothelial nitric oxide synthase, phosphorylated ERK, and phosphorylated Akt in the infarcted area early after acute myocardial infarction, leading to enhanced angiogenesis. Likewise, Hatanaka et [52] suggested a significant tendency to the activation of mechanosensors on cell membranes, such as caveolin-1 and β1-integrin, and subsequent phosphorylation of extracellular signal-regulated kinases (Erk) and protein kinase B (PKB/Akt) that may work a key role of the low-energy extracorporeal cardiac shock wave therapy to induce angiogenesis. Most recent studies that investigated the effect of shear stress are reported in Table 2

**Table 2:** Pivotal studies identifying the role of shear stress and perivascular adipose tissue. The references represent the progression in the text according to those reported in the PRISMA flowchart.

First Author/Year Ref	Type of Study	Cohort	Aims	Findings
Shindo et al. 2016 Arterioscler Thromb Vasc Biol [51]	Mouse model	Mouse Lipus Vs Mouse non Lipus	LIPUS implicated in amelioration of LV remodeling after IMA. Elucidate the underlying molecular mechanisms involved in the beneficial effects of LIPUS.	LIPUS therapy ameliorates post-myocardial infarction LV remodeling in mice in vivo. Increased vascular endothelial growth factor signaling
Hatanaka et al. 2016 Am J Physiol Cell Physiol [52]	Human model	HUVECs Vs HUVECs Knockdown of caveolin-1 or β1-integrin	Effects of SW irradiation on intracellular signaling pathways in vitro to induce myocardial angiogenesis	Activation of caveolin-1 and β1-integrin, and subsequent phosphorylation of Erk and Akt play crucial roles in the SW-induced angiogenesis.
Friederich-Persson et al. 2017 Arterioscler Thromb Vasc Biol [53]	Rat model	Wild-type Nox4^−/−^	Regulatory role and vasoprotective effects of BAT	BAT, via Nox4-derived hydrogen peroxide, induces cyclic GMP-dependent protein kinase G type-1α activation, resulting in reduced vascular contractility
Burgoyne et al. 2007 Science [54]	Mice model	SM22^+^, Nox5^+^, Nox5^+^/SM22^+ †^ WT	Whether concentration of oxydants in cells can regulate biochemical signaling mechanisms	Oxydants lead to cGMP-independent vasorelaxation in the cardiovascular system. H_2_O_2_ can operate as an endothelium-derived hyperpolarizing factor
Prysyazhna et al. 2012 Nat Med. 2012 [55]	Mice model	WT KI	Importance of PKGI-α oxidation in the EDHF mechanism and blood pressure control in vivo	C42S ‘redox-dead’ version of PKGI-α blocked the vasodilatory action of H_2_O_2_ on resistance vessels resulting in hypertension in vivo.
Noblet et al. 2015 Arterioscler Thromb Vasc Biol [56]	Suine model	Ossabaw swine obese vs. lean	Effects of lean and obese coronary PVAT on coronary vasodilation	Lean and obese coronary PVAT attenuates vasodilation via inhibitory effects on vascular smooth muscle K (+) channels. Calpastatin initiate or lead to progression of smooth muscle dysfunction in obesity.
Dou et al. 2017 Arterioscler Thromb Vasc [57]	Human model	AT-RAA (N = 74) AT-Mediastianal (n = 74)	AT-expressed ADAM17 activation in development of coronary microvascular dysfunction in obesity.	Aging and obesity decrease caveolin-1 expression. Increased vascular endothelial ADAM17 activity and soluble TNF release in AT
Candela et al. 2017 [58] Arterioscler Thromb Vasc	Mice model	Mice obese vs. lean	Role of macrophages in determining vascular [H_2_S] and vasodilatation	Vascular H_2_S depletion sustains the loss of perivascular adipose tissue anticontractile function in obesity.
Xia et al. 2016 Arterioscler Thromb Vasc Bio [59]	Mice model	C57BL/6J fat diet with or without PVAT	Contribution of PVAT to vascular dysfunction.	Diet-induced obesity leads to l-arginine deficiency and eNOS uncoupling in PVAT
Bussey et al. 2018 Arterioscler Thromb Vasc Biol [60]	Rat Model	Mesenteric arteries with and without PVAT from control	PVAT function after weight loss induced by caloric restriction	Diet-induced weight loss reverses obesity-induced PVAT damage due to reduced inflammation and increased nitric oxide synthase activity within PVAT

Abbreviations: Akt = protein kinase, AT = adipose tissue, ADAM17 = Tumour necrosis factor-α [TNF]-converting enzyme, BAT = brown adipose tissue, cGMP = guanosine 3′,5′-monophosphate, Cav-1 = caveolin-1, EDHF = Endothelium-derived hyperpolarizing factor, eNOS = endothelial NOS, Erk = extracellular signal-regulated kinase, H_2_O_2_ = hydrogen peroxide, H_2_S = hydrogen sulphide, HDF = high-fat diet, LIPUS = low-intensity pulsed ultrasound, LV = left ventricular, PKGIα = protein kinase G I-α, PVAT = perivascular adipose tissue, OS = reactive oxygen species, RAA = right atrial appendance, TNF = tumour necrosis factor, HUVEC = umbilical vein endothelial cell, WT = wild type.

### 3.3. Perivascular Adipose Tissue

Introduced as unexpected evidence in adult humans in 2007 [61], the vasoprotective action of perivascular adipose tissue (PVAT) has been rediscovered as crucial in controlling vascular function in the presence and absence of a pathological condition [62,63,64,65]. Studies investigating the protective or damaging effect of perivascular adipose tissue are shown in Table 2 [53,54,55,56,57].

**Figure 4:**
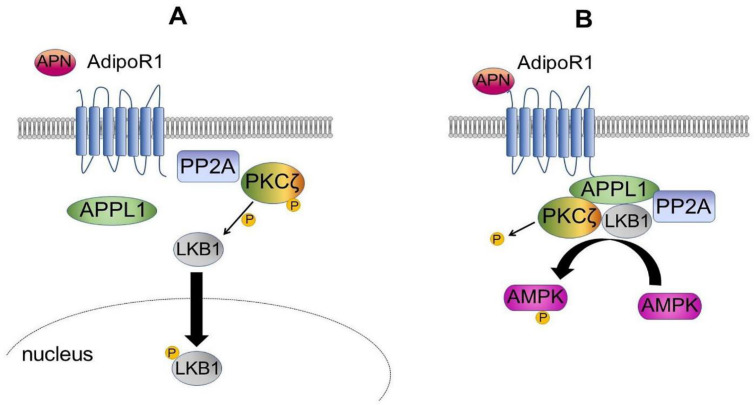
PVAT control vascular function by mean of precise mechanism related to adiponectin-dependent AMPK activation. Two conditions are relevant. (**A**) non-stimulated with no link between adiponectin (APN) and Adiponectin receptor 1 (AdipoR1). Adaptor protein, phosphotyrosine interacting with PH domain and leucine zipper 1 (APPL1) do not work alongside protein phosphatase 2A (PP2A). Protein kinase Cζ (PKCζ) induces phosphorylation of liver kinase B 1 (LKB1) affecting its translocation to the nucleus. (**B**) The stimulated state induces the bind of APN to AdipoR1 with activation of APPL1 resulting in PKCζ dephosphorylation by P. Dephosphorylated PKCζ is no longer effective to phosphorylate LKB1. PP2A also dephosphorylates LKB1, which results in LKB1 translocation to the cytoplasm. LKB1 phosphorylates AMP-activated protein kinase (AMPK) in the form of the AdipoR1/APPL1/PP2A/ PKCζ/LKB1 complex. Abbreviations are in the text.

The different pathophysiological role supported by PVAT allows its categorization as white, brown, and beige. This classification is related to its position in the organism with the purpose to regulate vascular tone in a paracrine/autocrine way allowing the release of a series of vasoactive substances, including NO, hydrogen sulphide, adiponectin, and others still to be identified [62]. The results emerged on the role offered by PVAT, which in physiological conditions is involved in powerful anti-atherogenic properties. The latter is mediated by its ability to secrete various biologically active factors which, thus, induce non-shivering thermogenesis and metabolize fatty acids. On the contrary, in the presence of specific pathological conditions, such as the state of obesity, PVAT becomes dysfunctional, losing its peculiar thermogenic capacity with the consequential effect of secreting pro-inflammatory adipokines that lead to endothelial dysfunction and infiltration of inflammatory cells, ultimately favouring the development of atherosclerosis [63] (Figure 4).

Recently, Hu et al. [64] reported that PVAT may be considered a supporting component of blood vessels, with a protective cushion effect for the vessel wall from the action of nearby tissues during relaxation and contraction. However, substantial evidence has emerged to support PVAT in the active regulation of blood vessel tone through vasoactive factors derived from PVAT itself, including relaxation and contraction factors. It should also be noted that PVAT contributes to atherosclerosis through the paracrine secretion of a large number of bioactive factors such as adipokines and cytokines. The observations received indicate that PVAT regulates blood vessel functions through various mechanisms that operate directly on the PVAT or underlying vessel layers, including vascular smooth muscle cells (VSMC) and EC. Starting from these findings, other studies can be undertaken to further investigate the actual contribution of the alterations in the metabolism of PVAT, which is responsible for the overall systemic outcomes determined by the alteration of its levels and the development of cardiovascular disease. This critical role remains largely unknown. In addition, special attention should be given to the role of the underlying messengers and mechanisms that are responsible for the crosstalk between PVAT and EC and VSMC in the vascular wall, as well as the contribution that PVAT aging can exert in vascular dysfunction [64].

The accumulated evidence on the function of perivascular adipose tissue emerged in one of the pivotal studies published to date and performed on a mouse model [53]. PVAT surrounding the aorta or mesenteric artery and interscapular brown adipose tissue showed a comparable role. Both work to ensure an anti-contractile effect through the activation of PKG1α (cyclic GMP-dependent protein kinase G1α) induced by H_2_O_2_ and the subsequent vasodilation of the arteries of low resistance [53], thus, explaining a therapeutic prospective of targeting brown adipose tissue in the cardiovascular disorders [54,55]. Likewise, in a recent study reporting impaired vasodilation on lean and obese coronary perivascular adipose tissue, researchers raised the hypothesis of a more sophisticated level of PVAT-mediated responses, using an ex vivo pig model. The effect of lean coronary PVAT supported a response towards the Kca and Kv7 channel-mediated vasodilatations. On the contrary, the opposed function of obese coronary PVAT revealed an impairment of Katp channel-mediated vasodilatation, thus suggesting the potential functions exerted by PVAT-derived factors in the pathogenesis of obesity-related coronary artery disease [56]. However, a substantial contribution to the understanding of the mechanism that supports the development of coronary microvascular dysfunction has been given by the findings on ADAM17. In the presence of aging and obesity, an increase of this disintegrin/metalloprotease molecule has been recorded. In this way, it has been shown to play a central role in the activity and release of soluble tumour necrosis factor in adipose tissue, leading to reduced endothelium-dependent vasodilation induced by bradykinin in human coronary arterioles [57].

It should be noted that obesity has detrimental effects on PVAT-mediated vascular function through the involvement of specific molecules, such as relaxing factors derived from the endothelium. As a first effect, it was observed that obesity raised the recruitment of proinflammatory macrophages into PVAT, favouring the impairment of the vasodilating property of PVAT. Lastly, a decrease in the endothelial and vascular levels of hydrogen sulphide produced by smooth muscle was highlighted, which has a powerful action as a gaseous relaxation factor [58]. A second action has been demonstrated and is related to diet-induced obesity. In this circumstance, a decoupling of eNOS levels from PVAT by arginase induced by L-arginine deficiency was revealed [59]. Although NOS3 has demonstrated its role in the generation of vasoprotective nitric oxide (NO), however, eNOS is not expressed exclusively in endothelial cells and recent studies have identified its expression in both adipocytes and PVAT endothelial cells. Therefore, PVAT eNOS has been shown to exert an important function that is included in the protective role of PVAT. Recently, Man et al. demonstrated that, in the presence of obesity-related metabolic diseases, PVAT eNOS may be even more important than the share of endothelial eNOS in promoting obesity-induced vascular dysfunction, which can ultimately be attributed to certain specific functions of PVAT eNOS [65]. Finally, in a third report, it was suggested that obesity-induced loss of the anti-contractile effect of PVAT was reversed after calorie restriction in a mouse model [60].

These findings open a window to the potential key therapeutic role of PVAT targeting in the treatment of cardiovascular disease associated with vascular dysfunction.

### 3.4. AMP-Activated Protein Kinase

Several recent reports have confirmed the remarkable role, previously described by Enkhjargal and colleagues, associated with AMP-activated protein kinase (AMPK) production in the treatment of metabolic disorders, including diabetes mellitus and obesity, in which vascular endothelial dysfunction is the main pathophysiological mechanism [66,67,68,69,70,71,72]. The role of AMP-activated protein kinase is well established in both the systemic and pulmonary circulation (Table 3).

The benefits associated with an increased level of endothelial AMP-activated protein kinase in the regulation of blood pressure and coronary flow responses were established in a landmark paper from Tohoku University [72]. Enkhjargal et al. used endothelium-specific AMPK knockout mice and in particular mice cohorts were composed by endothelium-specific deficiency of α-catalytic subunit of AMPK, either α1 (eAMPKα1 (−/−) α2 (+/+)) or α2 (eAMPKα1 (+/+) α2 (−/−)) alone, or both (eAMPKα1 (−/−) α2 (−/−)). Investigators found that the α1 subunit of endothelial AMPK was implicated in a central role in regulating blood pressure and coronary flow responses through EDH-mediated relaxations. However, the NO-mediated vasodilator response was not affected in mice cohorts. It is of note that in eAMPKα1 (−/−) α2 (−/−) cohort mice, antihypertensive treatment with hydralazine or long-term treatment with metformin is a stimulator of AMPK failed to restore EDH-mediated responses. The evidence provided the first direct effect of the α1 subunit of eAMPK that substantially was involved not only in EDH responses of microvessels, but also in the mechanism of regulation of blood pressure and coronary flow responses. Specifically, the findings offered two concepts. The first demonstrated the novel role of eAMPK in cardiovascular homeostasis. The second suggested that metformin is not only a drug of choice for the treatment of type 2 diabetes mellitus in the clinical setting but can also act as an activator of AMPK [72], as has recently been confirmed [73].

Cheang et al. [74], working in diet-induced obese PPARδ wild-type mice, revealed that the use of metformin reversed the aortic endothelial dysfunction by inhibiting endoplasmic reticulum stress caused by tunicamycin, oxidative stress, and impairment of endothelium-dependent relaxation. The induced ER stress was induced by the activation of the δ receptor pathway activated by the AMPK/peroxisome proliferator. Omura et al. [75], using a mice model, recently reported a significant influence of endothelial AMPK in protection against the development of pulmonary hypertension. In this study, the investigators found that metformin assumed a potential new role and could be a useful drug for the treatment of the disorder.

Similar to AMPK, antisenescence effects induced by sirtuin-1, which is an active protein against senescence, have been observed in endothelial cells and induced by cyclin-dependent kinase 5 (CDK5) that revealed a crucial role in the phosphorylation of SIRT1 at the serine 47 residue (S47) [76,77,78,79]. One of the main findings of the study revealed a severe senescence phenotype due to SIRT1phosphorylation at the S47 level in primary cultures of porcine aortic endothelial cells [78]. This reaction was significantly pronounced in the phenotype of endothelial cells predisposed to senescence. Significant evidence suggested the remarkable role of S47 phosphorylation, a process which was induced by agents that promote senescence and was attenuated by drugs with antisenescence properties. Researchers revealed that mutation of S47 to non-phosphorable alanine enhanced the evolution of cells towards senescence. Instead, the substitution of S47 with phosphoimiting aspartic acid abolished the anti-senescent, growth promoting, and downregulation actions of LKB1 by SIRT1 [79]. The role of CDK5 has been identified as crucial in modulating S47 phosphorylation of SIRT1 kinase. CDK5 knockdown or inhibition significantly decreased the number of senescent endothelial cells, promoted nuclear export of SIRT1, and attenuated the expression of inflammatory genes in porcine aortic endothelial cells [77].

A functional mechanism supported by the collaboration between AMPK and sirtuin-1 has been described as counteracting atherosclerosis in vivo mice models [80]. The atheroprotective effects underlying this process are initially mediated by the expression of the F-actin binding protein cortactin, which is coregulated by AMPK-induced phosphorylation. Subsequently, sirtuin-1 medicated deacetylation in rebound to the shear stress fostered the compartmentalisation and following activation of eNOS [80,81]. Additional research performed on a mouse model of induced diabetes has pointed out the function of endothelial microRNA-34a which can be upregulated by oxidative stress, thus leading towards endothelial dysfunction mediated by sirtuin-1 inhibition [82]. On the other hand, in a mouse model of induced hyperlipidemia, the activation of caspase-1 was inhibited during early atherogenesis, facilitating the accumulation of sirtuin-1 in endothelial cells with consequent anti-inflammatory effects [83]. From this evidence emerges a potential role of endothelial AMPK and sirtuin-1 as promising therapeutic targets for the treatment of cardiovascular diseases e metabolic disorders. Once more, further substantial data emerged from the study by Leung et al. which reported the contribution offered by sirtuin-1 and AMPK to endothelial functions by focusing on the key role of EDH-mediated responses in aging, hypertension, and sex difference [84].

### 3.5. Potassium Channels

The action exerted by the multitude of potassium channels in synergy with EDH, which sustains the vasodilation relaxation of vascular smooth muscle, plays a fundamental role in endothelial homeostasis for the support offered to the vasodilation processes [85,86,87,88,89,90] (Table 3).

The role of Kv7 channels in EPAC (exchange protein directly activated by cAMP) -dependent relaxations of the rat vasculature has been well established in a landmark paper of St George’s University Studies in the UK. The investigators working on a mouse model isolated rat renal and mesenteric arteries that were used for isometric tension to disclose the relaxant effects of a specific EPAC activator (8-pCPT-2Me-cAMP-AM) and the β-adrenoceptors agonist isoproterenol in the presence of potassium channel inhibitors and cell signalling modulators. They suggested KV7 channel-mediated relaxations in response to isoproterenol were contingent on a phenomenon of switch protein squarely operated by cAMP in the mesenteric artery, albeit this reaction was not evident in the renal artery. The reason for this difference may be the role played during the intermediate signalling steps from β-adrenoceptors to Kv7 channels which vary according to the vascular beds [85]. In renal arteries, it was noted that inhibitors of G protein βγ subunits (Gβγ) had decreased Kv7 activity and inhibited Kv7-dependent receptor-mediated vasorelaxations; however, the role of Gβγ in Kv7-dependent vasorelaxations of the rat vasculature was more recently evaluated [86]. The Gβγ function was analysed with gallein and M119K. The study revealed that isoproterenol increased association with Kv7.4 (potassium voltage-gated channel, KQT-like subfamily, member 4) and Ras-related protein (Rap1a) in the mesenteric artery, which was not sensitive to gallein, whereas, in the renal artery, isoproterenol increased Kv7.4-AKAP (A-kinase anchoring protein) associations in a gallein-sensitive manner. The findings proved that the Gβγ-Kv7 relationship differed between vessels and was an essential requirement for AKAP, but not Rap-mediated regulation of the channel [86]. Based on the knowledge that microtubules can regulate GPCR (G protein-coupled receptor) signalling in various cell types, Lindman and colleagues recently suggested that disruption of microtubules improved β-adrenoceptor-mediated relaxation of the mesenteric and renal arteries. Furthermore, the results that emerged demonstrated this improvement and determined that this improvement was due to the increased membrane expression of voltage-gated potassium channels Kv7 [87].

In a mouse model, it was reported that the blockade of KCa3.1 channels led not only to a reduction of the atherosclerotic burden, but also to improve the stability of the atherosclerotic plaque due to an inhibition in the differentiation towards the line of macrophages corresponding to the proinflammatory M1 phenotype [88]. Xu et al. [88] and Hu et al. worked a model for plaque instability that was induced by combined partial ligation of the left renal artery and left common carotid artery in apolipoprotein E knockout mice. In the first step investigators differentiated human monocyte into macrophages using macrophage colony-stimulating factor. The second step fixed that macrophages were polarized into proinflammatory M1 cells by interferon-γ and lipopolysaccharide and into alternative M2 macrophages by interleukin-4. During the differentiation of human monocytes into macrophages, a significant upregulation of KCa3.1 expression was disclosed. Blocking KCa3.1 significantly reduced the expression of proinflammatory genes during macrophages polarization. It is of note that in animal models, KCa3.1 blockade therapy surprisingly decreased the incidence of plaque rupture and luminal thrombus in carotid arteries, decreased the expression of markers associated with M1 macrophage polarization, and enhanced the expression of M2 markers within atherosclerotic lesions. These findings from advanced stages of atherosclerosis have magnified our knowledge of the critical role of macrophages on plaque instability, suggesting that KCa3.1 blockade suppresses plaque instability by inhibiting macrophage polarization towards an M1 phenotype [88].

However, it must be considered that the non-specific inhibition of KCa3.1, carried out without caution, could result in the opposite effect of causing endothelial microvascular dysfunction because it inhibits the EDH-mediated response [89,90]. Cardiovascular researchers have provided extensive explanations about the function of intermediate conductance KCa3.1 and low conductance KCa2.3 channels for their specific contribution to endothelium-derived hyperpolarization. For example, genetic deficiency of vascular K (+) channels results in severe impairments of local and systemic blood pressure regulation. These alterations reinforced the perspective that vascular K (+) channels are potential pharmacologic targets for improvement of vasodilator functions in cardiovascular pathologies [89].

Differently in the immunologist and neuroscience fields, these channels are mainly known for their part in lymphocyte activation and neuronal excitability. KCa3.1 is involved in the proliferation and migration of T cells, B cells, mast cells, macrophages, fibroblasts and dedifferentiated vascular smooth muscle cells and has, therefore, been widely considered as a potential target for use in some pathological conditions such as asthma, immunosuppression and in fibroproliferative disorders. On the contrary, the 3 KCa2 channels (KCa2.1, KCa2.2 and KCa2.3) have been associated with the phenomena of postperpolarization of the neuronal medium and therefore, depending on the type of neuron involved, worked in determining firing rates and frequencies or in regulating bursting. For this reason, KCa2 activators have been thoroughly investigated for potential therapeutic application in ataxia and epilepsy. Conversely, inhibitors of the KCa2 channel such as apamine are known to improve learning and memory in rodent [90]

**Table 3:** Most recent pivotal studies on vascular dysfunction and identifying the role of AMP-activated protein kinase, potassium channels, bone morphogenetic protein 4 and P2Y2 receptor. The references represent the progression in the text according to those reported in the PRISMA flowchart.

First Author/Year Ref	Type of Study	Cohort	Aims	Findings
Seki et al. 2017 J Am Heart Assoc [67]	Rat Model	SHRs with LCZ696 or valsartan Vs SHRs without LCZ696 or valsartan Vs WK	Wheter Angiotensin II receptor-neprilysin inhibitor sacubitril/valsartan. (LCZ696) would improve reduced EDH-mediated responses.	LCZ696 is effective as valsartan in improving the impaired EDH-mediated responses during hypertension. LCZ696 and valsartan exert beneficial effects on endothelium-independent relaxation by the ATP-sensitive K^+^ channel.
Jin et al. 2021 Sci Rep [71]	Rat model	HPH rats in vivo PMVEC in vitro	Whether CTRP9 has protective roles in the development of HPH.	CTRP9 lead to NO production and reduces ET-1 production by regulating AMPK activation. CTRP9 could be a target for HPH.
Omura et al. 2016 Circ Res. 2016 [75]	Mice model	eAMPK (−/−) eAMPK(flox/flox)	To determine the role of endothelial AMPK in the development of PAH.	Novel therapeutic target for the treatment of PAH exerted by endothelial AMPK.
Shentu et al. 2016 Arterioscler Thromb Vasc Biol [80]	Human model Mice model	Human umbilical vein ECs EC-specific AMPKα2	Whether AMPK and SIRT1 coregulate cortactin dynamics in response to shear stress.	AMPK/SIRT1 coregulated cortactin-F-actin dynamics is required for endothelial nitric oxide synthase. Atheroprotective role of AMPK/SIRT1.
Li et al. 2016 Arterioscler Thromb Vasc [82]	Mice model	db/db mice Infusion miR-34a inhibitor Genetic ablation of endothelial miR-34a	Role of endothelial miR-34a in diabetic vascular dysfunction by targeting Sirt1.	Endothelial upregulation of miR-34a leads to endothelial dysfunction by targeting Sirt1.
Stott et al. 2018 [85] Arterioscler Thromb Vasc	Rat model	RA vs. MA	The role of Kv7 channels in EPAC dependent relaxations of the rat vasculature.	EPAC-dependent vasorelaxations occur in part via activation of Kv7 channels. Effect in mesenteric, but not renal arteries.
Lindman et al. 2018 Ipertension [87]	In vitro rat model	WK RA Vs WK MA	The role of microtubule stability on β-adrenoceptor signaling in rat renal and mesenteric arteries.	Microtubule disruption improves the β-adrenoceptor-mediated relaxations of mesenteric and renal arteries.
Xu et al. 2016 [91] Arterioscler Thromb Vasc	Mice model	Apolipoprotein E knockout mice LRA Vs Apolipoprotein E knockout mice LCCA	Role of Kca3.1 I in macrophage polarization. Relationship to plaque instability.	Block of kCa3.1 suppresses plaque instability.
Hu et al. 2016 [91] Arterioscler Thromb Vasc	Mice model	Ad-Bmp4 Ad-Pdgfa-shRNA	Whether PDGF mediates BMP4-induced endothelial dysfunction in diabetes mellitus.	PDGF-AA impairs endothelium-dependent vasodilation. PDGF-AA mediates BMP4-induced adverse effect on endothelial cell function through SMAD1/5- and SMAD4-dependent mechanisms. Inhibition of PGDF-AA ameliorates vascular dysfunction.
Chen 2017 [92] Arterioscler Thromb Vasc	Mice model	EC-specific P2Y_2_R-deficient mice EC-specific P2Y_2_R knockout mice onto an ApoE^−/−^	Role of endothelial P2Y_2_R in the pathogenesis of atherosclerosis.	EC-specific P2Y_2_R deficiency reduces atherosclerotic burden and promotes plaque stability in ApoE^−/−^ mice.

Abbreviations: Ad-Bmp4, overexpress Bmp4; Ad-Pdgfa-shRNA, knockdown Pdgfa; AMPK, AMP-activated protein kinase; AMPKα2, EC-specific AMPKα2 knockout mice; ApoE^−/−^; knockout mice apoliprothein E; BMP4, Bone morphogenic protein 4; CTRP9, C1q/tumor necrosis factor-related protein-9; db/db; streptozotocine-induced diabetic mice; eAMPK(−/−), endothelial-specific AMPK-knockout mice; EC, endothelial cell; eAMPK(flox/flox, littermate control; EDH, endothelium-dependent hyperpolarization; EPAC, exchange protein directly activated by cAMP; HPH, hypoxia-induced pulmonary hypertension; KCa3.1, calcium-activated potassium channel type 3.1; Kv7, potassium channel type 7; LCZ696, receptor-neprilysin inhibitor sacubitril/valsartan; LCCA, left common carotid artery; LRA, left renal artery; MA, mesenteric artery; miR-34a, endothelial microRNA-34a; NO, nitric oxide; P2Y_2_R, Nucleotide P2Y_2_ receptor; PAH, pulmonary arterial hypertension; PDGF, platelet-derived growth factors; PMVEC; pulmonary microvascular endothelial cell; PKGIα, protein kinase G I-α; PVAT, perivascular adipose tissue; RA, renal artery; SHRs, spontaneously hypertensive rats; SIRT1, sirtuin 1; SMAD, Superfamily Mothers Against Decapentaplegic; WK, Wistar Kyoto.

### 3.6. Bone Morphogenic Protein 4

Several reports have substantially stated that BMP4 (bone morphogenic protein 4) plays a key role in the development of cardiovascular disease and in causing endothelial dysfunction in humans [91,93,94,95,96,97] (Table 3). A pivotal study by Wong and colleagues demonstrated that ROS served as a pathological link between BMP4 stimulation and the downstream cyclooxygenase-2 upregulation in endothelial cells, leading to endothelial dysfunction through ROS-dependent p38 mitogen-activated protein kinase activation. This BMP4/ROS/COX-2 cascade constituted a crucial function in the maintenance of endothelial dysfunction in hypertension [93]. Once more, Wong and colleagues found that BMP-4- or diabetes-mediated endothelial dysfunction was to a certain extent triggered by protein carbonylation reaction. The mechanisms that favour the blocking of this metal-catalysed protein oxidation may offer a new path toward an alternative therapeutic strategy capable of alleviating diabetic vasculopathy [94].

In more advanced studies, biologists learned that bone morphogenic protein 4 (BMP4) works as an important mediator of endothelial dysfunction in patients who develop cardiometabolic diseases and at the same time platelet-derived growth factors (PDGFs) interpret a crucial role as the most important angiogenic and proinflammatory mediators [91,95,96,97]. Therefore, the role of platelets is closely related to the function of BMP4. In a mixed model (human/mice) diabetes was induced by means of a generated Ad-Bmp4 to overexpress Bmp4 and Ad-Pdgfa-shRNA to obtain knockdown of Pdgfa in mice. Furthermore, the use of viruses, SMAD4-shRNA lentivirus, SMAD1-shRNA, and SMAD5 shRNA adenovirus, led to knockdown in human and mouse endothelial cells. The highlighted molecular mechanisms of endothelial dysfunction suggested the role played by BMP4 through binding to platelet-derived growth factors. Evidence significantly demonstrated that PDGF-AA impaired endothelium-dependent vasodilation. Furthermore, PDGF-AA mediated BMP4-induced adverse effects on endothelial cell function through SMAD1/5 and SMAD4-dependent mechanisms. The results unquestionably established that PGDF-AA inhibition improved vascular dysfunction in diabetic mice [91,95].

In a first study, Zhang and colleagues reported that Boldine was shown to be effective in reducing oxidative stress and improving endothelium-dependent relaxation in the aorta of diabetic mice mostly by inhibiting the overproduction of ROS associated with BMP4 mechanisms mediated by Angiotensin II [96]. In a second report, investigators demonstrated that the inhibition of BMP4 receptor/activin-like oxygen kinase 3/reactive species signalling resulted in an improved endothelial function in diabetic mice by limiting oxidative stress in the endothelium. Therefore, the inhibition of the BMP4 cascade may be an avenue to be pursued as a potential additional therapeutic strategy against diabetic vascular dysfunction [97].

### 3.7. P2Y2 Receptor

The incidence of serious issues due to a modification of atheromatous plaque can be significantly increased by endothelium-specific deletion of the P2Y2 receptor (Table 3).

With the use of a mouse model, it has been suggested that this receptor fulfilled substantial preventive effects on plaque stabilization by fostering the formation of the fibrous cap in atherosclerotic disorders [92]. Some considerations deserve a careful evaluation of the phenomena related to the relationship between the endothelial cell-specific P2Y2 and substances that exert vasodilatation such as acetylcholine. The occurrence of an endothelial P2Y_2_R deficiency decreased endothelial nitric oxide synthase activity alongside markedly modifying ATP- and UTP (uridine 5′-triphosphate) -induced vasorelaxation without affecting vasodilatory responses to acetylcholine. It is important to note that this evidence suggested that endothelial cell-specific P2Y2R deficiency decreased atherosclerotic cargo and contributed to plaque stability in ApoE^−/−^ mice. This process was sustained by an impaired macrophage infiltration acting together with decreased matrix metalloproteinase-2 activity and increased smooth muscle cell migration. Endothelial P2Y2 receptor may be a promising therapeutic target for atherosclerotic cardiovascular diseases [92,98,99].

Evidence showed that nucleotide P2Y_2_ receptor (P2Y_2_R) concur to the development of vascular inflammation by increasing vascular cell adhesion molecule-1 expression in endothelial cells (EC), and global P2Y_2_R deficiency prevents fatty streak formation in apolipoprotein E null (ApoE^−/−^) mice [98,99]. Chen et al. [92] investigated EC-specific P2Y_2_R-deficient mice that were engendered by breeding VEcadherin5-Cre mice with the P2Y_2_R floxed mice. The investigators found that endothelial P2Y_2_R deficiency decreased endothelial nitric oxide synthase activity and significantly altered ATP- and UTP (uridine 5′-triphosphate)-induced vasorelaxation without affecting vasodilatory responses to acetylcholine. Evidence suggested that EC-specific P2Y2R deficiency decreased atherosclerotic burden and promoted plaque stability in ApoE^−/−^ mice by promoting reduced macrophage infiltration. This action was simultaneous with the decreased activity of matrix metalloproteinase-2 [99] and the augmented migration of smooth muscle cells.

## 4. Endothelin and Cardiovascular Disease

Evidence has shown that aging is the primary risk factor responsible for the onset of CVD. As population growth estimates predict a doubling in the adult population over the age of 60 by 2050, controlling risk factors for CVD comorbidity and mortality becomes of paramount importance. Given that CVD prevalence and mortality rates have revealed a difference between men and women, an in-depth investigation into the biological processes of aging that evaluates specific mechanisms related to sex difference may be useful in studying vascular function The latter, in fact, offers substantial information on the health of the cardiovascular system [100,101]. Intervening changes in endothelial function generally precede changes in CVD prevalence rates in men and women. Furthermore, they are closely related to aging. The endothelin (ET) system plays a key role in the mechanisms that determine changes in endothelial function with a well-established link between NO Synthase and ET-1 [102,103,104,105,106]. In addition, there is a large body of discoveries that demonstrated the effects of sex and sex hormones on endothelin-1 (ET-1) and its ETA and ETB receptors. However, with advanced age a dysregulation occurs at the level of this system which leads to a consequent imbalance in the endothelial function between vasodilation and vasoconstriction. The observed dysregulation sees the role played by ET-1 as one of the main factors in vascular aging associated with impaired endothelial function among sex differences. Although most of the research has been conducted in preclinical animal models, evidence from more recent translational data in humans shows that the ET system is an important regulator of vascular dysfunction with aging and acts through sex-specific ET receptor mechanisms. Studies performed on the ET system have provided greater knowledge regarding the factors responsible for sex differences during vascular aging and endothelial dysfunction allowing for the use of optimized therapeutic strategies to mitigate the risk of CVD in the expanding aging population [107,108,109,110,111,112].

## 5. Conclusions

The emergence of recent experimental studies supported by clinical reports in the field of vascular biology have highlighted how a close relationship between endothelial dysfunctions and the onset of cardiovascular diseases is fundamental for the research and experimentation of new therapeutic approaches. Although there are still clear concerns about the complete knowledge of the mechanisms that regulate the modulation of endothelial functions, necessary for the improvement of clinical outcomes, nevertheless the vast literature published to date has recently revealed the emergence of crucial modulators of endothelial functions. The body of publications available today and discussed here offer new knowledge on cardiovascular diseases associated with the emergence of endothelial dysfunction, thus, opening a window on potential therapeutic and diagnostic targets with important clinical implications that ultimately make a fundamental contribution.

The complex role and function of the endothelium is vital for regular homeostasis; dysregulation may drive atherogenesis. Several factors have been postulated to play protective and deleterious effects in different individuals. Efforts should be placed at considering therapeutic options by targeting some of the factors noted to promote the protective properties of these agents. We are convinced that further characterization and better understanding of endothelial functions are certainly necessary to reach further milestones towards the application of new therapeutic strategies in cardiovascular medicine.

## Data Availability

Not applicable.

## Supplementary Materials

The following supporting information can be downloaded at: https://www.mdpi.com/article/10.3390/biomedicines10112884/s1, Table S1: PRISMA 2020 Checklist From: Page MJ, McKenzie JE, Bossuyt PM, Boutron I, Hoffmann TC, Mulrow CD, et al. The PRISMA 2020 statement: an updated guideline for reporting systematic reviews. BMJ 2021;372: n71. doi: 10.1136/bmj. n71

## Abbreviations

CABG	coronary artery bypass grafting
CAD	coronary artery disease
cGMP	cyclic guanosine monophosphate
EDH	endothelium-dependent hyperpolarization
EDRF	endothelium-derived relaxing factor
EC	Endothelial cell
H2O2	hydrogen peroxide
LITA	left internal thoracic mammary
NADPH	nicotinamide adenine dinucleotide phosphate
NO	nitric oxide
P2Y2	purinoceptor 2
PVAT	Perivascular Adipose Tissue
RCT	randomized clinical trial

Other abbreviations in tables and figures.
